# Erythropoiesis-Targeted Doping in Sports: From Improved Oxygen Transport to Cardiovascular Risk

**DOI:** 10.3390/pathophysiology33030062

**Published:** 2026-08-12

**Authors:** Gabriela Chlebowska, Krzysztof Michalak, Łukasz Mazur, Wioletta Szczurek-Wasilewicz

**Affiliations:** 1 Students’ Scientific Society, Department of Pharmacology, Faculty of Medicine, University of Opole, 45-052 Opole, Poland; 138971@student.uni.opole.pl; 2 Institute of Medical Sciences, Faculty of Medicine, University of Opole, 45-052 Opole, Poland; 127884@student.uni.opole.pl; 3 Institute of Chemistry, University of Opole, 45-052 Opole, Poland; lukasz.mazur@uni.opole.pl; 4 Department of Pharmacology, Faculty of Medicine, University of Opole, 45-052 Opole, Poland

**Keywords:** erythropoietin, erythropoiesis-stimulating agents, sports doping, blood doping, cardiovascular risk, Athlete Biological Passport, thromboembolism, gene doping

## Abstract

Erythropoiesis-targeted doping remains a major challenge for sports medicine because pharmacological and genetic manipulation of erythropoiesis can improve oxygen transport and endurance performance while increasing the risk of serious cardiovascular complications. Erythropoiesis-targeting strategies extend beyond recombinant erythropoietin (EPO) to include hypoxia-inducible factor prolyl hydroxylase inhibitors (HIF-PHIs), modified erythropoietin receptor (EPOR) agonists, transforming growth factor beta (TGF-β) signaling inhibitors, cytoprotective EPO derivatives, and gene- or cell-based approaches, and require complementary detection strategies based on direct analytical methods and the Athlete Biological Passport (ABP). Although they may enhance oxygen delivery and endurance performance, excessive stimulation of erythropoiesis may increase blood viscosity, impair vascular function, and elevate the risk of hypertension, thromboembolic complications, and other cardiovascular (CV) events. Erythropoiesis-targeted doping has evolved beyond recombinant EPO into a diverse group of pharmacological and genetic strategies that require increasingly sophisticated detection approaches. A thorough understanding of their molecular mechanisms and cardiovascular consequences is essential for improving anti-doping surveillance and protecting athlete health. This review summarizes current erythropoiesis-targeting agents, their mechanisms of action, detection strategies, cardiovascular risks, and implications for anti-doping practice.

## 1. Introduction

Following the introduction of recombinant human erythropoietin (rHuEPO) for the treatment of anemia in patients with chronic kidney disease (CKD) in the 1980s, its ability to increase the number of red blood cells (RBCs), hemoglobin (Hb) levels, and the blood’s oxygen-carrying capacity was quickly confirmed [1]. This same mechanism led to the misuse of rHuEPO preparations in sports, particularly in endurance disciplines such as cycling, long-distance running, and cross-country skiing. Experimental studies have shown that administration of rHuEPO can increase maximum oxygen uptake (VO_2_max) and improve selected parameters of physical performance [2,3]. According to the World Anti-Doping Agency (WADA) Prohibited List for 2026, erythropoietin (EPO) and other agents affecting erythropoiesis belong to Group S2.1. This category not only includes classic EPO preparations and their analogs, but also EPO mimetics and compounds that modulate the hypoxia-inducible factor (HIF) pathway, which is involved in the regulation of endogenous EPO production. In the context of doping targeting the erythropoietin–erythropoietin receptor (EPO–EPOR) axis, group M3, which encompasses gene and cellular doping, is also significant, as potential modifications of EPO expression, EPOR, or related signaling pathways could be used to increase erythropoiesis and improve oxygen transport capacity. According to the WADA Prohibited List, both substances from Group S2 and methods from Group M3 are prohibited both in-competition and out-of-competition [4]. Detecting doping related to the EPO–EPOR axis remains an analytical challenge. Classical methods for the direct detection of exogenous EPO, such as isoelectric focusing, sodium dodecyl sulfate-polyacrylamide gel electrophoresis (SDS-PAGE), and membrane-assisted isoform immunoassay (MAIIA), remain relevant but need to be supplemented with newer analytical and interpretive strategies [5]. For small molecules affecting erythropoiesis, including HIF activators, liquid chromatography coupled with tandem mass spectrometry (LC-MS/MS) plays a significant role. An additional monitoring tool is the Athlete Biological Passport (ABP), which allows for the assessment of changes in hematological parameters suggesting manipulation of erythropoiesis, although it does not replace methods that identify a specific substance [5]. Autologous or allogeneic red blood cell transfusion represents the simplest and most established method of increasing oxygen-carrying capacity, although transfusion-based blood doping is generally classified separately from gene and cell doping. For allogeneic transfusion, donor–recipient compatibility in the major ABO and Rhesus (Rh) blood group systems is essential; incompatibility may lead to severe hemolysis, renal failure, and, in extreme cases, death [6]. In recent years, interest has also grown in potential gene doping targeting the EPO pathway. Experimental methods for detecting exogenous deoxyribonucleic acid (DNA) encoding human EPO, as well as solutions based on clustered regularly interspaced short palindromic repeats/catalytically dead CRISPR-associated protein 9 (CRISPR/dCas9) and clustered regularly interspaced short palindromic repeats–CRISPR-associated protein 12a (CRISPR-Cas12a) technologies, have been described [7,8]. The possibility of detecting potential gene-doping agents containing transgenic DNA in the context of anti-doping testing has also been analyzed [9]. These data are currently of primarily developmental and analytical significance, but they demonstrate that the effectiveness of the anti-doping system will depend on the continuous adaptation of detection methods to new strategies of biological modification. The significance of EPO as a doping agent is not limited to improving oxygen transport and exercise capacity. Uncontrolled stimulation of erythropoiesis can lead to an increase in hematocrit (Ht), increased blood viscosity, and activation of the endothelium, platelets, and the coagulation system. These mechanisms may promote thromboembolic complications and increase the burden on the cardiovascular system, particularly under conditions of intense exercise, dehydration, or the concurrent use of other doping substances. For this reason, EPO abuse should be evaluated not only in terms of its ergogenic effect but also in terms of its potential cardiovascular and systemic consequences [5,10]. The aim of this review is to discuss the significance of EPO and other methods that stimulate erythropoiesis in sports doping, with particular emphasis on the mechanisms underlying improvements in physical performance, potential cardiovascular and systemic consequences, challenges related to detection, and the scale of the problem in both elite and amateur sports.

## 2. Methods—Search Strategy

The narrative review was conducted using targeted searches in the PubMed database, employing a combination of keywords related to erythropoiesis-based doping used by athletes and the cardiovascular (CV) system. The search included terms such as “doping,” “recombinant human erythropoietin,” “erythropoietin,” “genetic doping,” “darbepoetin alfa,” “pegmolesatide,” “roxadustat,” “hypoxia-inducible factor,” “anti-doping control,” “erythropoiesis-stimulating agent,” “transforming growth factor beta,” “Athlete Biological Passport,” “World Anti-Doping Code,” “athletes,” “prohibited substances,” “erythropoiesis,” “myocardial infarction,” “hypertension.” The cited literature covered the period from 1987 to 2026, with particular emphasis on current scientific evidence from 2000 onward.

High-quality evidence was considered first, with systematic reviews and meta-analyses, studies based on large cohorts and registries, randomized or pharmacodynamic studies where applicable, as well as the current 2026 guidelines of the WADA. Given the ongoing evolution of various forms of doping and methods for its detection, the review also includes preclinical studies and analytical studies. Where necessary, older, landmark publications were included to provide historical or mechanistic context.

Publications in languages other than English were not included. The review also includes individual case reports, which were included to highlight rare and severe complications of erythropoiesis-targeted doping; however, these were not a priority. In cases of conflicting evidence, priority was given to more recent studies and evidence-based recommendations.

## 3. Modern Forms of Doping Targeting Erythropoiesis

Modern doping strategies targeting erythropoiesis go beyond the use of classic recombinant EPO. They include modified EPOR agonists, EPO mimetics, and agents that modulate erythropoiesis via other biological pathways. According to the WADA classification, this group primarily includes EPOR agonists, HIF activators, GATA inhibitors, transforming growth factor beta (TGF-β) signaling inhibitors, and EPO derivatives that act as agonists of the so-called innate repair receptor (IRR) [4]. EPOR agonists include, among others, recombinant erythropoietins, darbepoetin alfa, methoxy polyethylene glycol-epoetin beta, a continuous erythropoietin receptor activator (CERA), EPO-Fc fusion constructs, and EPO mimetics such as CNTO-530, peginesatide, and pegmolesatide [4]. In anti-doping testing, compounds with prolonged duration of action, modified structure, or those that are difficult to detect using standard laboratory procedures are of particular significance.

### 3.1. EPOR Agonists and Modified EPO Derivatives

EPOR agonists include both classic recombinant erythropoietins and their modified derivatives, as well as mimetic molecules. Compounds with prolonged duration of action, greater biological activity, or altered structure are of particular significance for doping, as they can effectively stimulate erythropoiesis while simultaneously posing additional analytical challenges in anti-doping testing. Darbepoetin alfa is a modified EPO analog characterized by a prolonged half-life and greater in vivo biological activity than rHuEPO [11]. It is of significance in anti-doping testing due to its ability to stimulate erythropoiesis and the potential risk of abuse. Modified EPO derivatives also include CERA. Due to its modified structure and long-lasting effect, CERA represents a significant example of the development of erythropoietin preparations that go beyond classic rHuEPO. The low rate of CERA excretion in urine may limit the usefulness of this biological material for detection; therefore, blood analysis is also of significant importance in anti-doping testing [12,13]. Among the modified EPO-based constructs, fusion proteins such as EPO-Fc have also been described [14]. The combination of an EPO molecule with the fragment crystallizable (Fc) region of an immunoglobulin may affect pharmacokinetic properties, including prolonging the substance’s retention time in the body. In anti-doping testing, EPO-based fusion proteins are significant both because of their potential erythropoietic effect and the need to employ appropriate methods for their detection [14]. In addition to EPO analogs and modified derivatives, EPO mimetics have been developed. These molecules mimic EPO’s activity but have a different chemical structure. CNTO-530 is one example. In preclinical studies, Sathyanarayana et al. demonstrated that CNTO-530 induces a long-lasting erythropoietic effect in bone marrow proerythroblasts [15]. Pegmolesatide, on the other hand, is a long-acting EPO mimetic that activates EPOR as well as the Janus kinase 2/signal transducer and activator of transcription 5 (JAK2/STAT5) and extracellular signal-regulated kinases 1 and 2/mitogen-activated protein kinase (ERK1/2 MAPK) pathways [16]. The inclusion of such molecules in the list of prohibited substances demonstrates that modern erythropoietic doping encompasses not only exogenous forms of EPO but also structurally distinct compounds capable of activating the same biological pathway.

### 3.2. Hypoxia-Inducible Factor Prolyl Hydroxylase Inhibitors (HIF-PHIs)

HIF-PHIs are another group of substances that affect erythropoiesis. By stabilizing HIF, they enhance endogenous EPO synthesis and influence iron metabolism, which can lead to increased Hb levels and RBC mass. For this reason, despite their therapeutic use in treating anemia associated with CKD, they may have potential doping implications [17,18,19]. The best-known representative of this group is roxadustat, whose efficacy has been evaluated in patients with anemia associated with CKD, both non-dialysis and dialysis patients [16,19]. Unlike classic erythropoiesis-stimulating agents (ESAs), roxadustat does not directly activate EPOR but increases endogenous EPO production via the HIF pathway and improves iron availability for erythropoiesis [18,19]. In contrast to TGF-β signaling inhibitors, HIF-PHIs act through the physiological HIF–EPO–EPOR axis, whereas TGF-β inhibitors primarily enhance late-stage erythroid maturation without directly stimulating EPO production or EPOR signaling. Other members of this group include molidustat and vadadustat, which are also relevant from the perspective of anti-doping control [20,21].

### 3.3. TGF-β Signaling Inhibitors

Inhibitors of TGF-β signaling constitute another group of substances that influence erythropoiesis. The TGF-β superfamily includes, among others, activins, growth differentiation factors (GDFs), and bone morphogenetic proteins (BMPs), which can modulate the maturation of erythroid cells via the SMAD pathway [22]. Sotatercept is a glycosylated, dimeric fusion protein consisting of the extracellular domain of the human activin receptor type IIA (ActRIIA) and the Fc fragment of human immunoglobulin G1 (IgG1) [22]. This agent has demonstrated efficacy in treating pulmonary arterial hypertension (PAH) [23]. However, its influence on erythropoietic parameters suggests potential relevance in the context of doping. In a preclinical study, Carrancio et al. demonstrated that RAP-011, a mouse analog of sotatercept, increased Ht, RBC count, and Hb concentration [24]. Detection methods suitable for anti-doping control have also been developed for sotatercept, including techniques based on LC-MS/MS [25]. Luspatercept, formerly known as ACE-536, is a fusion protein composed of a modified extracellular domain of the type IIB activin receptor (ActRIIB) and the Fc domain of human IgG1 [26]. It promotes the late maturation of erythroid precursors through modulation of TGF-β signaling. In contrast to ESAs and HIF-PHIs, luspatercept and sotatercept promote late-stage erythroid maturation by inhibiting mothers against decapentaplegic homologs 2 and 3 (SMAD2/3) signaling rather than stimulating the HIF–EPO–EPOR axis. Their erythropoietic activity is therefore considered predominantly EPO-independent, although basal EPO signaling remains essential for normal erythropoiesis. To date, there is no convincing evidence that these agents directly activate the HIF–EPO axis in vivo. In clinical trials, luspatercept has demonstrated efficacy in the treatment of anemia associated with β-thalassemia, myelodysplastic syndromes, and primary myelofibrosis, primarily by reducing the need for RBC concentrate transfusions [26]. Their EPO-independent mechanism and ability to increase hemoglobin levels make both agents relevant from the perspective of anti-doping control.

### 3.4. EPO Derivatives with Cytoprotective Effects

The WADA classification includes specific EPO derivatives in Class S2, such as asialo-EPO and carbamylated erythropoietin (CEPO), which are identified as agonists of the IRR [4]. In contrast to classic rHuEPO, these compounds lack the typical EPOR-dependent erythropoietic effect but may preserve tissue-protective properties [27,28].

CEPO is generated by chemically modifying the EPO molecule, specifically through carbamylation of lysine residues. This alteration reduces its erythropoietic activity while maintaining cytoprotective properties [28]. In anti-doping control, the primary significance of these substances lies in the necessity to differentiate them from conventional forms of rHuEPO. For CEPO, a detection method involving enzymatic digestion of purified proteins with endoproteinase Lys-C has been described, which can supplement standard screening procedures in anti-doping laboratories [29].

### 3.5. Gene Doping

Recent advances in gene and cell therapies have enabled novel approaches for treating diseases caused by abnormalities in gene expression or function. However, these technologies also present the potential for misuse in enhancing athletic performance. Consequently, WADA prohibits gene and cell doping [4,7,30]. In gene therapy, targeting the genetic material of somatic cells is considered safer because the modifications are not inherited, unlike changes in germline cells, which result in heritable genetic changes. For doping targeting erythropoiesis, the ability to introduce the EPO gene into the body is of particular significance, as it can lead to excessive EPO expression, an increase in Ht, and enhanced oxygen transport to tissues. Potential gene-doping targets are not limited to EPO but may also include genes encoding HIF pathway regulators, erythropoietin receptor-related signaling molecules, and transcription factors involved in erythropoiesis and oxygen homeostasis. Direct in vivo delivery of EPO, or of another gene capable of stimulating erythropoiesis, to skeletal muscle or liver could conceivably be used to enhance performance. Animal models have shown that the transfer of the human EPO gene using viral vectors resulted in an increase in RBC count and an upregulation of hematopoiesis markers [31,32]. Adenoviral vectors, adeno-associated virus (AAV) vectors, and lentiviruses are most commonly used for this purpose. Transport can also occur via non-viral vectors, such as electroporation, plasmids, or related systems incorporating appropriate regulatory elements [30]. Recent advances in gene-editing technologies have expanded the spectrum of potential doping strategies beyond viral gene transfer. These include in vivo genome editing (e.g., CRISPR/Cas systems), antisense oligonucleotides, and microRNA (miRNA)-based approaches capable of modulating the expression of genes involved in erythropoiesis, muscle adaptation, and recovery, although their application for performance enhancement remains largely theoretical at present. Alternatively, cell-based doping could involve implantation of engineered EPO-secreting cells, transplantation of encapsulated engineered cells, or transfer of genetically modified muscle progenitors. In these approaches, the transduced or implanted cells would secrete EPO into the circulation without requiring marrow ablation. Animal studies have demonstrated that intramuscular or intravenous gene delivery can increase circulating EPO levels and Ht [31,32]. Gene- or cell-based approaches are significantly less invasive. It should be emphasized here that there are methods aimed at hematopoietic stem cell transplantation (HSCT) or ex vivo genetic modification of hematopoietic stem cells (HSCs). HSCT is primarily used to treat hematologic cancers and, increasingly, autoimmune diseases [33]. However, it is extremely rare in the context of doping. HSCT carries a risk of serious adverse effects (AEs). It requires the use of intensive myeloablative therapy and sometimes total-body irradiation. The main causes of HSCT-related deaths are infections, cytopenias, and cardiotoxicity. A sufficiently long follow-up period and recovery period are also necessary [34]. All of this means that athletes very rarely opt for HSCT and instead use less invasive methods based on viral vectors and cell-based approaches. At the same time, methods have been developed to detect gene doping based on the analysis of DNA and ribonucleic acid (RNA) derived from whole blood [30,31,32]. Current human-based detection strategies focus primarily on identifying vector-specific DNA sequences, transgene-derived DNA or RNA, and abnormal gene expression profiles in blood samples using polymerase chain reaction (PCR) and sequencing-based methods. CRISPR-based diagnostic assays have also emerged as promising tools because of their high analytical sensitivity and sequence specificity. However, they remain experimental and have not yet been implemented in routine WADA-accredited anti-doping laboratories [30]. Gene doping raises significant safety concerns. Potential risks include uncontrolled gene expression, excessive erythropoiesis, thromboembolism, immune reactions, and, with certain vectors, insertional mutagenesis. From the perspective of anti-doping control, detecting traces of genetic manipulation remains a particular challenge, especially when the biological effect persists longer than the vector, or when the transgene is no longer detectable in readily accessible biological samples [7,31,32]. Beyond the analytical challenges, gene doping raises important ethical and legal concerns because it undermines fair competition while exposing athletes to interventions with uncertain long-term safety. Consequently, WADA prohibits both gene transfer and gene-editing approaches intended to enhance athletic performance. The characteristics of each substance are presented in Table 1.

## 4. Detection of Erythropoietic Doping: Direct Methods, Biological Passport, and Interpretive Limitations

Detecting doping related to the EPO–EPOR axis remains a significant analytical challenge. Direct methods, such as isoelectric focusing, SDS-PAGE, or immunological techniques, allow for the identification of exogenous forms of EPO but have limitations. Interpreting direct tests for exogenous EPO can be difficult because kidney disease or intense physical exertion can alter urine composition and lead to atypical analytical results [40]. In contrast, the SDS-PAGE and MAIIA methods require prior purification of urine or blood samples using immunoaffinity, which increases the technical and organizational demands of the test [41,42]. For small molecules, such as HIF stabilizers or EPO mimetics, LC-MS/MS plays a crucial role. These analytical methods facilitate the detection of compounds that are challenging to identify using classical protein-based techniques. However, a key limitation is the requirement for suitable reference materials, which are not always readily available [43]. Direct detection methods are supplemented by the ABP, which was introduced by WADA in 2009. The ABP relies on long-term monitoring of individual hematological parameters, including Hb concentration, Ht, RBC count, reticulocytes, and RBC indices [5,44]. Indirect measures, such as the OFF-score and the abnormal blood profile score (ABPS), can be calculated from these parameters to detect abnormalities indicative of erythropoiesis manipulation [5,45]. The biological passport enhances the sensitivity of anti-doping testing, especially in cases involving microdosing or substances with a short detection window. However, it does not replace analytical methods that identify specific substances [5,44]. Direct numerical comparison of sensitivity and specificity across the available detection methods remains difficult because the available approaches differ in analytical targets, study designs, and interpretive thresholds [44,46,47]. Interpretation of ABP results requires caution, as hematological parameters may fluctuate due to factors such as training, dehydration, altitude exposure, seasonality, illness, and individual variability [45,46,47]. These influences can complicate the assessment of an athlete’s biological profile and increase the risk of misinterpretation [46]. False-positive findings may result from physiological or pathological conditions, such as altitude exposure, dehydration, illness, or renal disorders, whereas false-negative results may occur after microdosing or delayed sample collection. In practice, these limitations are minimized by combining direct analytical methods with longitudinal ABP monitoring and expert interpretation of clinical and training-related data. To reduce the risk of false-positive interpretations related to physiological variations such as altitude exposure, the ABP uses an adaptive Bayesian statistical model based on each athlete’s longitudinal hematological profile rather than fixed population-based thresholds. Nevertheless, abnormal ABP findings should always be interpreted alongside clinical information, training history, altitude exposure, and complementary analytical evidence [47].

## 5. Detectability in the Anti-Doping System Versus the Estimated Scale of Doping Use

Anti-doping testing data demonstrate that agents affecting erythropoiesis continue to be a primary focus of monitoring. WADA report on 2024 anti-doping test results documented 61,663 urine sample analyses and 5723 blood sample analyses for erythropoietin receptor agonists (ERAs). Adverse analytical findings (AAFs) were identified in 149 samples [48]. Relative to 2015, the number of ERA tests increased substantially, from 32,999 to 61,663 for urine samples and from 3219 to 5723 for blood samples. Concurrently, the number of AAFs rose from 46 in 2015 to 149 in 2024 [48]. In its press release on the 2024 report, WADA emphasized both the increased volume of ERA testing and the corresponding rise in AAFs within this category. It is essential to recognize that official detection rates do not equate to the actual prevalence of EPO use, its derivatives, or other methods influencing erythropoiesis. An AAF denotes a laboratory result indicating the presence of a prohibited substance or another abnormal parameter in a biological sample. However, AAFs do not account for undetected cases, use outside the detection window, manipulations that are challenging to identify, or behaviors that do not result in a positive test result. This limitation is particularly relevant for doping practices targeting oxygen transport, as certain methods may primarily induce indirect alterations in the hematological profile.

### Survey Studies Based on the Randomized Response Technique

Survey studies and indirect analyses suggest that the scale of doping use may be greater than would be indicated solely by official laboratory statistics. It should be emphasized, however, that most survey studies do not assess the use of EPO or specific ERAs separately. Most often, the general prevalence of the use of prohibited substances and methods, or broader categories such as peptide hormones, blood doping, or blood manipulation, is analyzed. Ulrich et al. assessed the prevalence of doping among 2167 athletes participating in two major sporting events: the 13th World Athletics Championships in 2011 and the 12th Pan Arab Games in Doha. The authors used the randomized response technique (RRT), which allows for answering sensitive questions while maintaining greater anonymity. The estimated prevalence of doping use in the past year was 43.6% during the World Championships and 57.1% during the Pan-Arab Games [49]. In the same study, the official results of biological testing were significantly lower: 0.5% positive results during the World Championships and 3.6% during the Pan-Arab Games [49]. These data do not refer exclusively to EPO, but they clearly illustrate the limitations of assessing the scale of doping based solely on the results of anti-doping tests. In addition, because the RRT included questions about all prohibited methods or substances, it was not possible to determine what proportion of these cases specifically involved erythropoiesis-stimulating agents. Analyses based on hematological parameters are more specifically targeted at blood doping. Faiss et al. estimated the prevalence of blood doping among international-level track and field athletes during two major international competitions based on hematological parameters in a study that was divided into individual, anonymized subgroups of countries. The estimated prevalence of blood doping was 18% in 2011 and 15% in 2013 [50]. While this is not a direct measure of EPO use, these results indicate that manipulations aimed at improving oxygen transport may be more common than positive doping test results alone suggest. Similarly, Sottas et al. described the collection of 7289 blood samples from 2737 athletes during an international track and field competition. Endurance athletes were specifically targeted for testing, as approximately 79% of samples were obtained from athletes competing in events of 800 m or longer. While differences between endurance and nonendurance athletes were anticipated, nationality emerged as the primary source of heterogeneity. The estimated prevalence of blood doping ranged from 1% to 48% across subpopulations, with a mean prevalence of 14% for the entire study population. Nationality data were anonymized to prevent any attribution of results to specific countries [51]. The collection of reliable, detailed data on doping among athletes, classified by sport and region, as well as the determination of the percentage of cases involving the use of erythropoiesis-stimulating agents, remains a research gap.

The important matter is that data from RRT surveys are not equivalent to data from official statistics. For this reason, they should not be considered as directly comparable [48,49]. The RRT method refers to the most recent 12-month period during which an athlete self-reported doping violations via a questionnaire [49]. In contrast, official WADA statistics reflect urine or blood samples in which an AAF was detected [48]. The reasons for this discrepancy are multifactorial. Drug tests often prioritize specificity over sensitivity, which can result in false-negative outcomes and an underestimation of true doping prevalence due to insufficient sensitivity [50,51]. Furthermore, the use of low dosages of doping substances by athletes leads to brief detection windows [50,52]. Certain tests, such as those conducted during competitions or at random, may primarily serve as deterrents rather than effectively identifying doping cases in real time. Although athlete surveys offer a potential alternative, obtaining truthful responses from elite athletes, who may seek to avoid suspicion, remains challenging [49,50].

Survey data covering athletes subject to anti-doping testing also indicate that categories related to peptide hormones and blood manipulation appear in athletes’ self-reports, although less frequently than the general use of prohibited substances. In a study by Davoren et al., involving 1398 American athletes subject to testing in accordance with WADA regulations, the overall estimated prevalence of doping was 6.5–9.2%. In terms of specific categories, 0.4% of respondents reported using peptide hormones, 0.6% reported blood manipulation to enhance performance or aid recovery, and 0.1% reported methods involving stem cells or gene editing [53]. These data do not allow for an unambiguous determination of the prevalence of EPO use, but they indicate that biological and hematological methods should be analyzed as a separate risk group.

The problem of doping is not limited to elite sports. Dietz et al. conducted an anonymous survey using the RRT technique among a group of 2997 amateur triathletes participating in competitions in Germany. The estimated prevalence of doping use over the past 12 months was 13.0% [54]. These results indicate that the use of performance-enhancing substances or methods may also affect individuals who train and compete at the amateur level, especially in endurance sports, where the potential benefit of increased oxygen transport is particularly significant. More recent survey data also confirm that the use of prohibited substances or methods may be reported by some high-level athletes. It should be emphasized, however, that survey studies are subject to response bias, underreporting, and methodological differences across populations.

A comparison of WADA data with survey results and hematological analyses reveals a significant discrepancy between official detection rates and the estimated scale of doping use. In the case of EPO, its derivatives, and other methods affecting erythropoiesis, this discrepancy is particularly significant, as detection may depend on the timing of sample collection, the type of substance used, dosing strategies, and the sensitivity of analytical methods. Therefore, a more comprehensive assessment of the scale of the problem requires combining laboratory data, an ABP, hematological analyses, and survey data, in both elite and amateur sports.

## 6. Mechanism of Action of EPO and Erythropoiesis-Stimulating Agents

Erythropoietin is a glycoprotein cytokine that regulates the homeostasis of the erythropoietic system. Its expression is strictly dependent on tissue oxygen availability and is controlled primarily by the HIF pathway [55,56]. Under conditions of normal tissue oxygenation, the activity of this pathway is suppressed, and EPO gene expression remains at a low level. Hypoxia, on the other hand, leads to the stabilization of hypoxia-inducible factor alpha (HIF-α), its translocation to the cell nucleus, and the activation of the hypoxia-responsive transcriptional program, which includes, among others, the gene encoding EPO [55,56]. This process results in increased EPO production, primarily in the kidneys, which stimulates erythropoiesis and enhances the blood’s ability to transport oxygen.

The biological action of EPO depends on its binding to the EPOR, which is primarily present on erythroid progenitor cells in the bone marrow. EPOR belongs to the family of cytokine receptors and does not exhibit its own kinase activity. Upon ligand binding, a conformational change in the receptor occurs, leading to the activation of JAK2, the phosphorylation of EPOR’s cytoplasmic domains, and the activation of signaling pathways responsible for the proliferation, differentiation, and survival of erythroid cells [57,58,59]. The best-characterized EPO-dependent cascade is the JAK2/STAT5 pathway. Activated STAT5 undergoes dimerization, translocates to the cell nucleus, and regulates the expression of genes governing the survival and maturation of progenitor cells. Of particular importance is the induction of anti-apoptotic genes, including the BCL2 like 1 (BCL2L1) gene, which encodes the B-cell lymphoma-extra large (Bcl-xL) protein; this protein inhibits apoptosis in erythrocyte precursors and enables their further differentiation [57,58]. Concurrently, the phosphoinositide 3-kinase/protein kinase B (PI3K/AKT) and mitogen-activated protein kinase/extracellular signal-regulated kinase (MAPK/ERK) pathways are activated. The PI3K/AKT pathway supports cell survival, metabolism, and the anti-apoptotic response, whereas the MAPK/ERK pathway is primarily involved in regulating the proliferation and expansion of erythroid cells [59]. EPO promotes the survival, proliferation, and terminal differentiation of erythroid progenitors, particularly colony-forming unit-erythroid (CFU-E) cells and early erythroblasts. Through these effects, EPO indirectly increases the production of mature red blood cells, hemoglobin concentration, and the blood’s oxygen-carrying capacity [57,58,59].

Of particular importance are HIF-PHIs such as roxadustat, daprodustat, molidustat, and vadadustat [4,60,61,62]. These substances pharmacologically mimic hypoxia by stabilizing HIF, which leads to increased endogenous EPO production and upregulation of genes involved in iron metabolism [60,61]. Unlike conventional rHuEPO preparations, their mechanism of action does not involve the direct administration of exogenous EPO, but rather the modulation of the intracellular hypoxia response system. For this reason, HIF-PHIs pose a significant challenge from the perspectives of anti-doping control and the assessment of potential health consequences. The ergogenic effect of rHuEPO stems primarily from an increase in the blood’s oxygen-carrying capacity, but it should not be interpreted solely as a simple increase in the number of RBCs. Administration of rHuEPO to healthy individuals leads to an increase in total Hb mass and, in some studies, also to improvements in performance parameters such as peak oxygen uptake (VO_2_peak), time to exhaustion, or time trial performance [63,64,65,66]. The magnitude of this effect depends on the dose, duration of use, level of training, sex, exercise protocol, and the endpoint used [63,64,66]. However, interpreting VO_2_max or VO_2_peak as a single indicator of doping effectiveness requires caution, since in elite athletes, performance in endurance disciplines also depends on movement economy, lactate threshold, maximum power, tolerance for submaximal exercise, muscle adaptations, and the ability to maintain exercise intensity over time [3,64,67].

Annaheim et al. demonstrated that, following rHuEPO administration, the improvement in time to exhaustion may be greater than the increase in VO_2_max, suggesting the involvement of mechanisms beyond mere improvement in oxygen transport [3]. The authors pointed, among other things, to the potential effects of EPO on skeletal muscle metabolism, central fatigue, the vascular response, and lactate concentration during submaximal exercise [3]. Some of the effects of rHuEPO may also occur before a full increase in erythrocyte mass is achieved. A reduction in plasma volume (PV), changes in water and electrolyte balance, and a possible effect on the renin–angiotensin–aldosterone system (RAAS) have been reported, which may temporarily increase Hb and Ht concentrations independently of a full increase in RBC mass [65,68,69,70].

**Figure 1:**
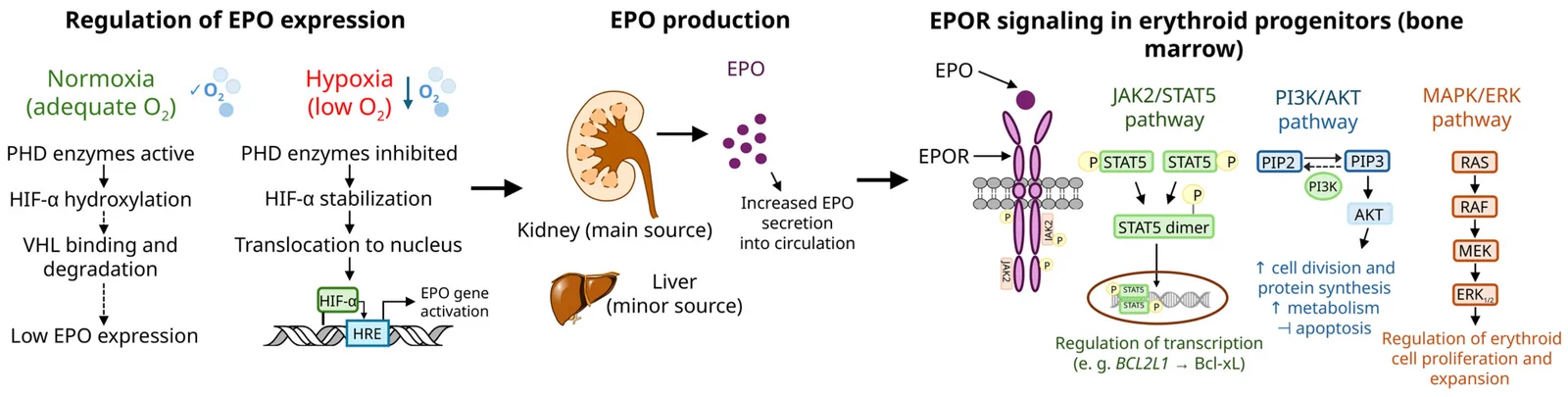
Mechanisms of action of erythropoietin and erythropoiesis-stimulating agents. AKT—protein kinase B; BCL2L1—BCL2 like 1; Bcl-xL—B-cell lymphoma-extra large; EPO—erythropoietin; EPOR—erythropoietin receptor; ERK—extracellular signal-regulated kinase; HIF-α—hypoxiainducible factor alpha; HRE—hypoxia-response element; JAK2—Janus kinase 2; MAPK—mitogen-activated protein kinase; MEK—mitogen-activated protein kinase; P—phosphate group; PHD—prolyl hydroxylase domain; PI3K—phosphoinositide 3-kinase; PIP2—phosphatidylinositol 4,5-bisphosphate; PIP3—phosphatidylinositol 3,4,5-trisphosphate; RAF—rapidly accelerated fibrosarcoma; RAS—rat sarcoma virus; STAT5—signal transducer and activator of transcription 5; VHL—von Hippel–Lindau.

The mechanism of action of ESAs should therefore be understood as a multilevel intervention in the physiological hypoxia–HIF–EPO–EPOR axis, as shown in Figure 1. The end result is an increase in erythrocyte mass and oxygen-carrying capacity and simultaneously a modification of the signaling pathways responsible for cell survival, iron metabolism, the response to hypoxia, PV regulation, and exercise adaptation. This framework provides a better explanation of both the potential ergogenic effect and the biological basis for the AEs associated with the non-medical use of EPO and other ESAs.

## 7. Cardiovascular Consequences of EPO Doping

The cardiovascular consequences of the misuse of EPO and other ESAs result from simultaneous interference with oxygen transport, blood rheological properties, vascular tone regulation, endothelial function, platelet activity, and the coagulation system [5,10,71,72,73,74]. An increase in RBC mass and Hb concentration improves the blood’s ability to transport oxygen; however, it may simultaneously impair blood flow. An increase in Ht raises blood viscosity, impedes microcirculatory flow, increases vascular resistance, and may increase afterload on the heart. During intense exercise, the significance of these changes is further amplified, as dehydration, hyperthermia, and sympathetic nervous system activation can reduce PV, increase relative Ht, and heighten the myocardium’s oxygen demand [5,72,73]. A key risk factor is the effect of EPO on the vascular endothelium. The endothelium regulates vascular tone, platelet activity, leukocyte adhesion, and the balance between coagulation and fibrinolysis. Under physiological conditions, vasodilatory and anticoagulant mechanisms predominate, depending among other factors, on nitric oxide (NO) and prostacyclin. Excessive or non-medical stimulation of EPO may shift this balance toward vasoconstriction, endothelial activation, and increased procoagulant potential. A reduction in NO bioavailability may play a significant role, accompanied by an intensification of vasoconstrictive mechanisms dependent on endothelin, angiotensin II, prostanoids, and increased sympathetic activity [73]. In a study involving trained cyclists, administration of rHuEPO was associated with increased levels of E-selectin and P-selectin, suggesting endothelial activation and increased platelet reactivity [10]. These changes may promote leukocyte adhesion, platelet activation, and a shift in the hemostatic balance toward a prothrombotic state. This effect may be particularly significant during prolonged exercise, dehydration, hyperthermia, and intense adrenergic activation, which are conditions commonly observed in endurance sports [10].

### 7.1. Thromboembolism

There is little direct population-based data in the available literature assessing the incidence of myocardial infarction, stroke, or venous thromboembolism in athletes who abuse EPO. This is due to the covert nature of doping, difficulties in determining the dose and duration of exposure, and the frequent concomitant use of other substances. Case reports indicate, however, that the non-medical use of EPO may be associated with severe thromboembolic complications, including cerebral venous sinus thrombosis and acute coronary syndrome in young athletes [74,75]. In contrast, clinical trials using ESAs in patients with chronic diseases suggest that achieving higher Hb levels may increase the risk of serious cardiovascular events, particularly stroke and thrombotic complications. Although these data cannot be directly extrapolated to healthy athletes, they support the biological plausibility of the risks associated with excessive stimulation of erythropoiesis [71].

### 7.2. Blood Pressure

EPO may contribute to elevated blood pressure through mechanisms beyond increased Ht and blood viscosity. A critical factor is the shift in vascular balance toward vasoconstriction, characterized by reduced NO bioavailability, increased endothelin activity, and heightened vascular responsiveness to catecholamines and angiotensin II [73]. Consequently, peripheral resistance may rise, imposing a greater hemodynamic burden on the heart. Lundby et al. demonstrated that prolonged administration of rHuEPO resulted in increased systolic blood pressure both at rest and during exercise, indicating that the ergogenic effect is accompanied by an increased hemodynamic load on the cardiovascular system [76].

### 7.3. Multifactorial Cardiovascular Effects

The cardiovascular risk associated with EPO doping is therefore multifactorial. Clinical evidence suggests that cardiovascular risk increases with higher ESA doses and higher achieved Hb concentrations, although this dose–response relationship has not been established in healthy athletes. In doping practice, microdosing regimens are commonly used to reduce detectable hematological changes while maintaining ergogenic effects. Although microdosing may attenuate acute increases in blood viscosity, its cardiovascular safety remains uncertain. It results from an imbalance between increased oxygen transport, vascular flow, and the hemostatic system. Increases in Ht and blood viscosity, activation of the endothelium and platelets, heightened prothrombotic potential, elevated blood pressure, and increased vascular reactivity may mutually reinforce one another. In this context, substances that improve oxygenation may simultaneously increase the risk of stroke, acute coronary syndrome, venous thromboembolism, and sudden cardiovascular events, especially when used off-label and without monitoring of hematological parameters [5,10,71,72,73,74].

## 8. Other Health Consequences of EPO Use

In addition to cardiovascular risks, the misuse of EPO and other ESAs can lead to hematologic, immunologic, neurological, and systemic complications. A particular concern is the use of preparations from unregulated sources, which may vary in actual active ingredient content, purity, stability, dosage, and immunogenicity profile. In clinical settings, the use of ESAs requires monitoring of Hb and Ht levels, blood pressure, and iron metabolism parameters. In sports doping, such monitoring typically does not occur, which increases the risk of unpredictable AEs [5,77].

### 8.1. Anti-EPO Antibody-Mediated Pure Red Cell Aplasia

One rare but potentially severe complication associated with ESA use is anti-EPO antibody-mediated pure red cell aplasia. This mechanism involves the development of neutralizing antibodies that may cross-react with endogenous EPO, leading to inhibition of erythropoiesis and severe anemia [78]. The risk of such a reaction is of particular concern with preparations of uncertain quality, those stored improperly, or those used without medical supervision and monitoring of hematological parameters [78,79].

### 8.2. Neurological Consequences

Possible neurological and vascular complications should also be considered, particularly in cases of elevated blood pressure and impaired autoregulation of cerebral circulation. Cases of hypertensive posterior leukoencephalopathy associated with the use of rHuEPO have been described in the literature [79]. Although these reports are rare, they indicate that the consequences of EPO abuse may extend beyond the cardiovascular system to include the central nervous system as well. The risk of such complications may be higher with high or uncontrolled doses, concomitant increases in Ht, dehydration, and lack of blood pressure monitoring.

### 8.3. Tissue Effects

EPO also exhibits pleiotropic effects that go beyond the classic stimulation of erythropoiesis. Experimental studies have described its effects on cell survival, the inflammatory response, oxidative stress, apoptosis, the mobilization of endothelial progenitor cells, and the processes of angiogenesis and tissue repair [27,56,80,81,82,83,84,85]. Some of these effects may result from EPO’s action beyond the classical erythropoietic axis, including the modulation of immune and endothelial cell activity [27,56]. However, the significance of these observations in the context of doping remains unclear, as most of the data come from experimental studies or patient populations, rather than from healthy athletes using EPO for non-medical purposes.

### 8.4. Limitations

From a safety perspective, it is crucial to distinguish between the controlled use of ESAs in medicine and their non-medical use to enhance performance. Clinical indications require monitoring of the therapeutic effect and the risk of AEs, whereas doping involves uncertainty regarding dose, duration of exposure, product quality, and the concurrent use of other substances. Therefore, EPO abuse should be viewed not only as an attempt to increase oxygen transport but also as a potential source of hematological, immunological, neurological, and systemic complications [5,77,78,79].

## 9. Conclusions

Doping targeting the EPO–EPOR axis remains one of the most significant challenges in modern anti-doping efforts. It not only encompasses classic recombinant EPO preparations, but also modified EPOR agonists, EPO mimetics, HIF-PHIs, TGF-β signaling inhibitors, cytoprotective EPO derivatives, and genetic and cellular strategies. The development of these methods indicates that erythropoietic doping is no longer limited to increasing Hb and Ht levels, but may also involve more complex modulation of biological pathways responsible for erythropoiesis, oxygen transport, tissue protection, and regeneration.

A significant challenge remains the detection of new substances, particularly those with altered structures, a short detection window, or those used in microdosing regimens. Therefore, classical methods of direct detection must be supplemented with highly specialized techniques, such as LC-MS/MS, and long-term assessment of hematological parameters as part of an ABP. The results of these methods require careful interpretation, as blood parameters can be influenced by training, dehydration, exposure to high altitude, seasonality, and the athlete’s health status.

Improving ABP interpretation should not rely solely on revising existing thresholds. Instead, longitudinal adaptive modeling should be combined with candidate complementary biomarkers, including messenger RNA (mRNA) expression of 5′-aminolevulinate synthase 2 (ALAS2) and carbonic anhydrase 1 (CA1), the immature reticulocyte fraction (IRF), and the immature-reticulocyte-to-red-blood-cell ratio (IR/RBC). Interpretation should also account for individual and contextual factors, including PV shifts, sex, iron status, training, dehydration, altitude exposure, seasonality, and health status.

Official anti-doping testing statistics primarily reflect the detectability of doping within a specific testing system, rather than its actual prevalence among the athlete population. Data from WADA reports should therefore be compared with the results of anonymous surveys, which suggest that the scale of use of prohibited substances and methods may be greater than indicated by the number of AAFs.

The use of substances and methods that affect erythropoiesis may be associated with significant health risks, particularly with an uncontrolled increase in red blood cell mass and blood viscosity. The most significant risks include thromboembolic complications, hypertension, and other cardiovascular events. The impact of newer forms of erythropoietic doping on athletes’ health remains not fully understood, particularly when used outside of medical indications, without clinical supervision, and with technologies whose long-term effects are difficult to predict.

These findings also highlight the need for comprehensive anti-doping prevention strategies that combine advanced analytical testing with educational initiatives targeting athletes, coaches, and medical personnel. Furthermore, integrating analytical data with behavioral surveillance approaches, including anonymous prevalence surveys, may provide a more accurate assessment of doping practices and support the development of more effective evidence-based anti-doping policies.

## Figures and Tables

**Table 1:** Classification, mechanisms of action, pharmacokinetic properties, and anti-doping relevance of erythropoiesis-targeting agents.

Class	Example	Mechanism/Characteristics	Route of Administration	Plasma Half-Life (t_1_/_2_)	Anti-Doping Detection/Relevance	References
EPOR agonists and modified EPO derivatives	Darbepoetin alfa	Second-generation ESA; hyperglycosylated recombinant EPO analogue; ↑ EPOR–JAK2/STAT5 pathway activity, resulting in ↑ erythropoiesis and ↑ hemoglobin levels	SC/IV	≈21–25 h (IV) ≈48–73 h (SC)	Detectable by SAR-PAGE and IEF; urinary detection generally persists for several days, whereas hematological effects may persist for weeks	[11,12,35]
	Methoxy polyethylene glycol-epoetin beta (CERA)	Third-generation ESA; PEGylated continuous erythropoietin receptor activator; ↑ erythropoiesis and Hb via EPOR activation	SC/IV	≈120 h (IV) ≈124 h (SC)	Long elimination half-life prolongs the detection window; detected by IEF/SAR-PAGE with LC-MS-based confirmatory analysis	[13,35,36]
	EPO-Fc	Fc-fusion protein consisting of recombinant human EPO linked to the Fc domain of IgG1; ↑ EPOR–JAK2/STAT5 signaling → ↑ erythropoiesis and hemoglobin	SC/IV	Not established; markedly prolonged by FcRn-mediated recycling	Experimental compound; no validated WADA-specific detection assay currently available	[14,35]
	CNTO-530	Synthetic Fc-fusion protein (mimetibody) with no sequence homology to EPO; ↑ EPOR–JAK2/STAT5 signaling → prolonged stimulation of erythroid progenitors	SC	≈48–72 h (preclinical studies)	Experimental ESA; prolonged erythropoietic activity may complicate indirect detection	[15]
	Pegmolesatide	PEGylated 44-amino-acid synthetic peptide lacking homology to endogenous EPO	SC/IV	≈30–50 h	Synthetic peptide ESA; detectable by peptide-specific LC-MS/MS methods	[16]
HIF-PHIs	Roxadustat	Orally active, reversible small-molecule HIF-PHD inhibitor; stabilizes HIF → ↑ endogenous EPO, ↓ hepcidin, ↑ iron availability and erythropoiesis	Oral	≈10–16 h	Detected in urine by LC-MS/MS; prohibited at all times (WADA S2)	[18,20,21,37]
	Molidustat		Oral	≈4–6 h	Short plasma half-life; detectable in urine by LC-MS/MS	
	Vadadustat		Oral	≈5–8 h	Detected by targeted LC-MS/MS	
Inhibitors of TGF-β signaling	Sotatercept	Recombinant ActRIIA-Fc fusion protein that acts as a ligand trap for members of the TGF-β superfamily; ↑ late-stage erythroid maturation (↓ SMAD2/3 signaling) ↑ Hb (EPO-independent); approved for PAH	SC	≈24 days	Long biological activity; detectable in serum by immunoblotting and LC-HRMS	[23,24,25,35,38]
	Luspatercept	Recombinant ActRIIB-Fc fusion protein acting as a ligand trap for selected TGF-β superfamily members; ↓ SMAD2/3 signaling → ↑ late-stage erythroid maturation and hemoglobin (EPO-independent); approved for anemia associated with MDS and BT	SC	≈11 days	Long biological activity; hematological effects may persist for weeks, potentially affecting longitudinal hematological monitoring	[26,35,39]
	Carbamylated EPO	Chemically modified EPO derivative that does not bind the classical EPOR homodimer; activates the EPOR–β-common receptor heteroreceptor, promoting cytoprotection and anti-inflammatory effects	SC/IV (experimental studies)	Not established (short in experimental models)	No erythropoietic activity; limited relevance for current anti-doping testing	[28,29]
EPO derivatives with cytoprotective activity	Asialoerythropoietin	Desialylated EPO derivative; no clinically relevant erythropoietic effect; retains tissue-protective and cytoprotective activity	IV	≈2–5 min	Very short detection window due to rapid clearance; included among prohibited EPO derivatives by WADA	[4,27,28]
Gene doping	-	Transfer of genes (e.g., EPO) or gene-editing technologies (e.g., CRISPR/Cas) to enhance athletic performance; ↑ endogenous EPO production and/or activation of pathways involved in oxygen transport, muscle growth, or recovery; long-lasting biological effects with substantial health risks	Gene transfer	Not applicable	Difficult to detect; monitored using molecular assays and indirect biomarkers	[30,31,32]

ActRIIA—activin receptor type IIA; ActRIIB—activin receptor type IIB; BT—beta-thalassemia; CERA—continuous erythropoietin receptor activator; CRISPR/Cas—clustered regularly interspaced short palindromic repeats/CRISPR-associated proteins; EPO—erythropoietin; EPOR—erythropoietin receptor; ESA—erythropoiesis-stimulating agent; Fc—fragment crystallizable; FcRn—neonatal Fc receptor; Hb—hemoglobin; HIF—hypoxia-inducible factor; HIF-PHI—hypoxia-inducible factor prolyl hydroxylase inhibitor; IEF—isoelectric focusing; IgG1—immunoglobulin G1; IV—intravenous; JAK2—Janus kinase 2; LC-HRMS—liquid chromatography–high-resolution mass spectrometry; LC-MS—liquid chromatography–mass spectrometry; LC-MS/MS—liquid chromatography–tandem mass spectrometry; MDS—myelodysplastic syndromes; PAH—pulmonary arterial hypertension; PEG—polyethylene glycol; PHD—prolyl hydroxylase domain; SAR-PAGE—sarcosyl polyacrylamide gel electrophoresis; SC—subcutaneous; SMAD2/3—mothers against decapentaplegic homologs 2 and 3; STAT5—signal transducer and activator of transcription 5; TGF-β—transforming growth factor beta; WADA—World Anti-Doping Agency.

## Data Availability

No new data were created or analyzed in this study. Data sharing is not applicable to this article.

## Abbreviations

The following abbreviations are used in this manuscript:

AAF	Adverse analytical finding
AAV	Adeno-associated virus
ABP	Athlete Biological Passport
ABPS	Abnormal blood profile score
ActRIIA	Activin receptor type IIA
ActRIIB	Activin receptor type IIB
AE	Adverse effect
AKT	Protein kinase B
ALAS2	5′-aminolevulinate synthase 2
BCL2L1	BCL2 like 1
Bcl-xL	B-cell lymphoma-extra large
BMP	Bone morphogenetic protein
CA1	Carbonic anhydrase 1
CEPO	Carbamylated erythropoietin
CERA	Continuous erythropoietin receptor activator
CFU-E	Colony-forming unit-erythroid
CKD	Chronic kidney disease
CRISPR	Clustered regularly interspaced short palindromic repeats
CV	Cardiovascular
dCas9	Catalytically dead CRISPR-associated protein 9
DNA	Deoxyribonucleic acid
EPO	Erythropoietin
EPO–EPOR	Erythropoietin–erythropoietin receptor
EPOR	Erythropoietin receptor
ERA	Erythropoietin receptor agonist
ERK	Extracellular signal-regulated kinase
ESA	Erythropoiesis-stimulating agent
Fc	Fragment crystallizable
GDF	Growth differentiation factor
Hb	Hemoglobin
HIF	Hypoxia-inducible factor
HIF-α	Hypoxia-inducible factor alpha
HIF-PHI	Hypoxia-inducible factor prolyl hydroxylase inhibitor
HSC	Hematopoietic stem cell
HSCT	Hematopoietic stem cell transplantation
Ht	Hematocrit
IgG1	Immunoglobulin G1
IR	Immature reticulocyte
IRF	Immature reticulocyte fraction
IRR	Innate repair receptor
JAK2	Janus kinase 2
LC-MS/MS	Liquid chromatography–tandem mass spectrometry
MAIIA	Membrane-assisted isoform immunoassay
MAPK	Mitogen-activated protein kinase
miRNA	MicroRNA
mRNA	Messenger RNA
NO	Nitric oxide
PAH	Pulmonary arterial hypertension
PCR	Polymerase chain reaction
PI3K	Phosphoinositide 3-kinase
PV	Plasma volume
RAAS	Renin–angiotensin–aldosterone system
RBC	Red blood cell
Rh	Rhesus blood group system
rHuEPO	Recombinant human erythropoietin
RNA	Ribonucleic acid
RRT	Randomized response technique
SDS-PAGE	Sodium dodecyl sulfate-polyacrylamide gel electrophoresis
SMAD2/3	Mothers against decapentaplegic homologs 2 and 3
STAT5	Signal transducer and activator of transcription 5
TGF-β	Transforming growth factor beta
VO_2_max	Maximum oxygen uptake
VO_2_peak	Peak oxygen uptake
WADA	World Anti-Doping Agency
